# Ultrasonographic modeling of diaphragm function: A novel approach to respiratory assessment

**DOI:** 10.1371/journal.pone.0229972

**Published:** 2020-03-12

**Authors:** Danielle N. O’Hara, Andrey Pavlov, Erin Taub, Sahar Ahmad

**Affiliations:** 1 School of Medicine, Stony Brook University Hospital, Stony Brook, New York, United States of America; 2 Department of Medicine, Division of Pulmonary and Critical Care Medicine, Stony Brook University Hospital, Stony Brook, New York, United States of America; 3 Department of Biostatistics, Stony Brook University Hospital, Stony Brook, New York, United States of America; University of Notre Dame Australia, AUSTRALIA

## Abstract

**Objectives:**

Bedside ultrasound techniques have the unique ability to produce instantaneous, dynamic images, and have demonstrated widespread utility in both emergency and critical care settings. The aim of this article is to introduce a novel application of this imaging modality by utilizing an ultrasound based mathematical model to assess respiratory function. With validation, the proposed models have the potential to predict pulmonary function in patients who cannot adequately participate in standard spirometric techniques (inability to form tight seal with mouthpiece, etc.).

**Methods:**

Ultrasound was used to measure diaphragm thickness (Tdi) in a small population of healthy, adult males at various points of the respiratory cycle. Each measurement corresponded to a generated negative inspiratory force (NIF), determined by a handheld meter. The data was analyzed using mixed models to produce two representative mathematical models.

**Results:**

Two mathematical models represented the relationship between Tdi and NIF_max_, or maximum inspiratory pressure (MIP), both of which were statistically significant with p-values <0.005: 1. log(NIF) = -1.32+4.02×log(Tdi); and 2. NIF = -8.19+(2.55 × Tdi)+(1.79×(Tdi^2^)).

**Conclusions:**

With validation, these models intend to provide a method of estimating MIP, by way of diaphragm ultrasound measurements, thereby allowing evaluation of respiratory function in patients who may be unable to reliably participate in standard spirometric tests.

## Introduction

Evaluation of respiratory function is a vital component in the assessment of patients with a wide range of clinical derangements. Spirometric techniques, such as negative inspiratory force (NIF) and vital capacity (VC), are commonly used for such assessments. These techniques, however, are limited by patients’ ability to form a tight seal with their lips around a measurement device, or to coordinate precise instructions from a respiratory technician [1]. Alternative assessment via ultrasound (US) imaging of the diaphragm presents a possible method for alleviating such limitations.

US has demonstrated a wide utility in emergency and critical care medicine [2–5], serving as a non-invasive imaging modality that produces real-time visualization of physiologic and pathologic processes. Owing to its ability to produce instantaneous, dynamic images, US can be used to observe diaphragm thickening during inspiration [6]. Previously, US measurements recorded at the diaphragm’s Zone of Apposition (ZOA) have shown to correlate with the muscle’s strength and shortening [7], and have been further proven to reliably assess global diaphragm function [6, 8–10].

Establishing a mathematical relationship between maximum NIF, or maximum inspiratory pressure (MIP), a value derived by standard spirometric techniques, and diaphragm thickness may permit an assessment of patients with various respiratory ailments, such as unexplained dyspnea or recurrent respiratory failure, through imaging alone. This would be particularly meaningful in those with neuromuscular conditions, for example, whereby a lack of cooperation or facial muscle weakness impede reliable NIF measurements [1, 11]. Our study explored a mathematic correlation between diaphragm thickness and MIP in healthy adults.

## Materials and methods

This study was performed in accordance with the Declaration of Helsinki. This human study was approved by Stony Brook University Human Subjects Committee (IRB)—approval: #716520–3. All adult participants provided written informed consent to participate in this study.

Diaphragm thickness was measured in ten self-reported healthy male adults in a seated position by placing a linear array high frequency probe at a right lateral scan plane between the 8^th^ and 10^th^ intercostal spaces. One ultrasound machine was used by one study administrator to measure and record all data values. Measurements were exclusively performed on the right side, in accordance to prior descriptions in the literature of this technique and due to the impediment of accurate imaging and measurement due to bowel gas and splenic density seen on the left. The location of the diaphragm’s ZOA was confirmed by the presence of lung artifacts (eg, lung sliding, A-lines) approaching the image during inspiration. With the display set to B-mode, diaphragm thickness (Tdi) was measured from inside edge of the pleural line to the inside edge of the peritoneal line. Measurements for each volunteer were recorded in duplicate at the end of tidal expiration, representing functional residual capacity (FRC), and during generated NIF of -30 cm of H_2_O, -60 cm H_2_O, and maximum NIF, which is also known as maximum inspiratory pressure (MIP). NIF measurements were recorded by a handheld NIF meter.

Statistical analysis was conducted using SAS Institute Inc. v9.4 (NC, Cary). A scatterplot showing the relationship between measured NIF and absolute diaphragm thickness (Tdi) was built. Two representative mathematical models were generated using a mixed model, with individual subjects as the random effect, to account for repeated measures. All measured NIF values were adjusted by adding 0.1 to produce non-zero values to permit logarithmic transformation.

To create a linear model, both the *x* and *y* variables were log transformed to account for the non-linearity of the measured data. A mixed model was generated using logarithmic-transformed variables to validate the following model:

log(NIF)=‐1.32+4.02×log(Tdi) (1)

A mixed model using the original, untransformed data was used to validate the following model, which follows a quadratic function:

$$\mathrm{NIF}=‐8.19+(2.55\times \mathrm{Tdi})+(1.79\times ({\mathrm{Tdi}}^{2}))$$ (2)

## Results

Ten healthy 20-30-year-old males were included in this study as a convenience sample. The mean (SD) Tdi at NIF_0_, or at FRC, was 1.21 (0.34) mm and increased to a mean (SD) of 7.38 (1.03) mm at MIP, when the diaphragm is thought to be maximally contracted [7]. The mean (SD) MIP was 126.6 (3.27) cm H_2_O.

**Fig 1:**
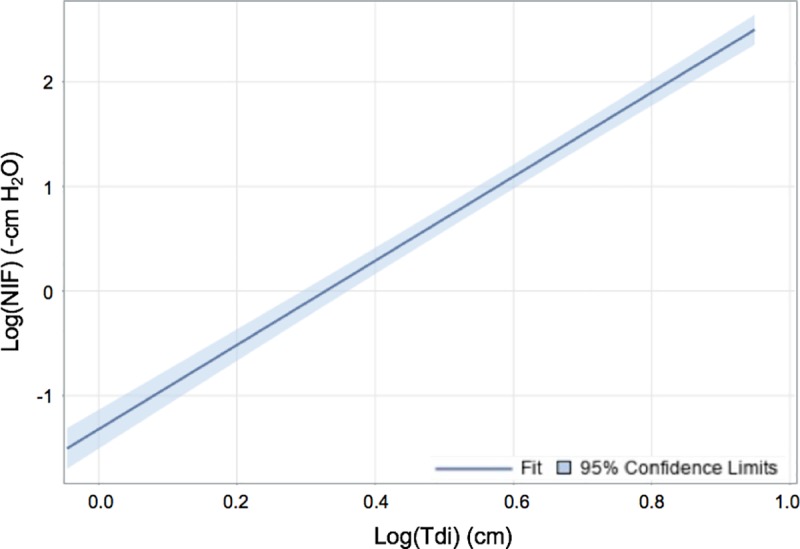
Scatter plot and fitted line of the linear regression model with logarithmic transformation of NIF versus the absolute diaphragm thickness (Tdi). The best-fit line is represented by y = -1.32 + 4.02× log(x). This mixed model using logarithmic transformation of *x* and *y* variables demonstrated a p-value <0.0001.

**Fig 2:**
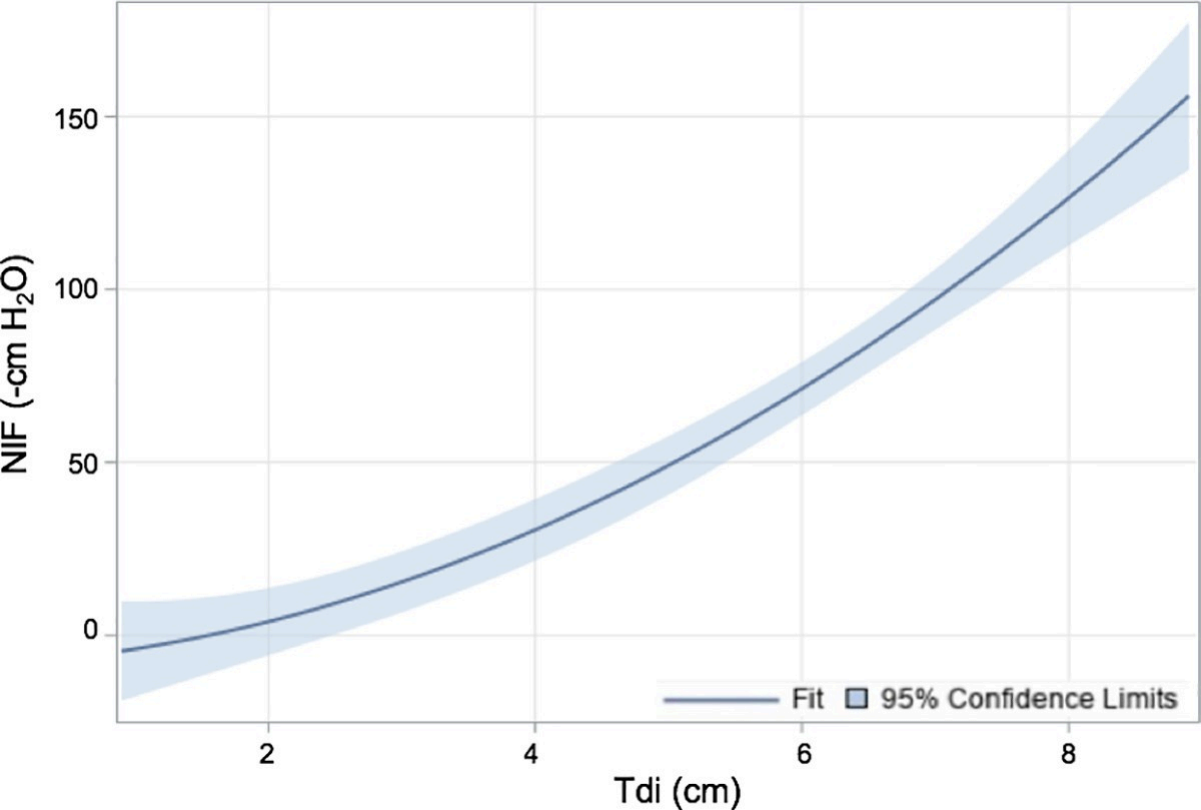
Scatter plot and fitted line of the general linear model used to represent the quadratic relationship between NIF and Tdi. The best-fit line is represented by y = -8.19 + (2.55 × Tdi) + (1.79 × (Tdi^2^)). This mixed model of the original untransformed data demonstrated a p-value <0.005.

This study demonstrates a mathematic correlation between Tdi and MIP as represented by models (1) and (2). A linear function describes the correlation between log-transformed values of Tdi and NIF as shown in Fig 1. A quadratic function describes the correlation between measured Tdi and NIF as shown in Fig 2. These relationships are considered significant, with p-values <0.005. The intraclass correlation coefficient was 0.298, indicating that mean Tdi values among subjects were similar, but individual subjects’ Tdi values differed among generated NIF values. Taken together in this study population, these models show that US can provide an objective, reliable, and reproducible measurement of diaphragm function, and it has the potential to predict patients’ MIP.

## Discussion

The models produced in this study elucidate the mathematical relationship between diaphragm thickness and NIF_max_, also known as the maximum inspiratory pressure (MIP). MIP is a measure of global respiratory muscle strength and, as such, is closely related to diaphragm strength [1]. MIP has been shown to detect respiratory muscle weakness before other spirometric measures, such as forced vital capacity (FVC) and total lung capacity (TLC) [1, 12]. This is a valuable feature, as respiratory weakness can lead to respiratory failure and early mortality [13, 14], and it is associated with the need for noninvasive or invasive ventilation to prolong survival [1]. In fact, respiratory failure that occurs secondary to respiratory muscle weakness is a common cause of premature death in patients with neuromuscular disorders [1].

Although MIP measurements may be able to detect respiratory muscle weakness sooner than other assessment methods, it has a high number of false negatives because it is a volitional test [11]. Volitional tests of respiratory muscle strength rely on patient factors such as the level of coordination and the ability to form a tight seal with their lips [11, 15]. Therefore, it is of paramount importance to incorporate non-volitional tests into the clinical assessment of patients with suspected respiratory muscle weakness [11].

As it currently stands, transdiaphragmatic pressure is a non-volitional test that serves as the gold standard for measuring diaphragm strength; however, this method is invasive and requires an experienced practitioner [11]. US of the diaphragm presents an alternative non-volitional test that is non-invasive and easily accessible, produces no patient discomfort, and can be performed rapidly with good repeatability [11, 15].

Our study utilized a small population of healthy adult males to produce two mathematical models that demonstrate the correlation between diaphragm thickness and MIP. Model (1) may produce more accurate estimations of patients’ MIP, whereas model (2) provides a simpler calculation which may be more useful for bedside predictions.

Our study is limited by the small convenience sample size and the fact that all our subjects are young and healthy adult males. Similarly, the low intraclass correlation (ICC) can be attributed to the homogenous sample and small number of subjects. Due to the fact that one examiner performed all measurements, the results only reflect this specific rater involved in the study. The ICC is only in the level of indicating a fair reliability [16]; further validation is needed to inform clinical utility of these models. Therefore, the models produced in this study cannot be directly applied to the evaluation of broader patient populations without validation in further studies that include a larger group of male and female patients with a wide range of ages and health statuses, including those with and without neuromuscular disorders.

With validation in future studies, the models produced in this study have the potential to predict MIP based on ultrasound measurements. This method is independent of patient factors that may otherwise limit MIP measurements, such as the ability to form a tight seal at the lips or follow the respiratory technician’s instructions, or in those with a depressed mental or neurological status. Therefore, these models exhibit potential for objectively delineating a patient’s respiratory function.

## Supporting information

S1 Dataset Study dataset. (XLSX)
